# Implementation of cryogenic tender X-ray HR-XANES spectroscopy at the ACT station of the CAT-ACT beamline at the KIT Light Source

**DOI:** 10.1107/S1600577521012650

**Published:** 2022-01-01

**Authors:** Bianca Schacherl, Tim Prüssmann, Kathy Dardenne, Kirsten Hardock, Volker Krepper, Jörg Rothe, Tonya Vitova, Horst Geckeis

**Affiliations:** a Institute for Nuclear Waste Disposal (INE), Karlsruhe Institute of Technology (KIT), Hermann-von-Helmholtz-Platz 1, 76344 Eggenstein-Leopoldshafen, Germany

**Keywords:** KIT light source, CAT-ACT beamline, ACT station for radionuclide research, high-resolution X-ray emission, actinide *M*-edge HR-XANES, cryogenic measurements

## Abstract

The ACT experimental station of the CAT-ACT wiggler beamline at the Karlsruhe Institute of Technology (KIT) Light Source is dedicated to the investigation of radionuclide materials with radioactivities up to 1000000 times the exemption limit by various speciation techniques applying monochromatic X-rays. In this article, the latest technological developments at the ACT station that enable high-resolution X-ray absorption near-edge structure (HR-XANES) spectroscopy for low radionuclide loading samples are highlighted – encompassing the investigation of actinide elements down to 1 p.p.m. concentration – combined with a cryogenic sample environment reducing beam-induced sample alterations. One important part of this development is a versatile gas tight plexiglass encasement ensuring that all beam paths in the five-analyzer-crystal Johann-type X-ray emission spectrometer run within He atmosphere. The setup enables the easy exchange between different experiments (conventional X-ray absorption fine structure, HR-XANES, high-energy or wide-angle X-ray scattering, tender to hard X-ray spectroscopy) and opens up the possibility for the investigation of environmental samples, such as specimens containing transuranium elements from contaminated land sites or samples from sorption and diffusion experiments to mimic the far field of a breached nuclear waste repository.

## Abstract 2

The capabilities for the investigation of radionuclide materials by high-resolution X-ray emission spectroscopy techniques at the CAT-ACT beamline of the Karlsruhe Institute of Technology (KIT) Light Source have been expanded to allow for the investigation of redox-labile low-concentration samples at cryogenic temperatures.

## 1. Introduction

Synchrotron radiation based speciation methods for radioactive samples are often limited to dedicated beamline endstations due to the strict safety and radiation protection regulations for handling radionuclide materials at light sources (Scheinost *et al.*, 2021 ▸). Two of those few endstations – the INE-Beamline and the ACT station at the Karlsruhe Institute of Technology (KIT) Light Source (Rothe *et al.*, 2012 ▸; Zimina *et al.*, 2017 ▸) – are operated by KIT-INE (the Institute for Nuclear Waste Disposal at KIT). However, some synchrotron facilities accept proposals to investigate radioactive materials on conventional beamlines if the radionuclide activities stay below the exemption limit. Others temporarily upgrade the safety status of selected endstations to allow for radionuclide research up to a certain extent – generally excluding highly radioactive materials like spent nuclear fuel or certain isotopes. During the past decade, high (energy) resolution X-ray absorption near-edge structure (HR-XANES) spectroscopy – also called high-energy-resolution fluorescence-detected X-ray absorption near-edge structure (HERFD-XANES) – has proven to be a highly valuable tool for the oxidation state determination of actinides (An). However, in this case, on the one hand the inefficient detection process requires high photon fluxes (where samples might be prone to beam-induced alterations) and rather high analyte concentrations. On the other hand, it is necessary to find a trade-off between necessary sample containment and radiation safety precautions at synchrotron radiation facilities and sufficiently transparent X-ray windows. Hence, already for investigations at the moderately high X-ray energies corresponding to the An *L*_3_ absorption edges (Th–Es: ∼16.3–20.4 keV), HR-XANES investigations of actinides are experimentally highly demanding. This applies all the more in the ‘tender’ X-ray regime below ∼4.5 keV, where the corresponding An *M* absorption edges are probed. It is for this reason, for example, that HR-XANES data for the *L*- and *M*-level absorption edges of transuranium elements within the An family basically stem from only a few X-ray emission spectrometers at four synchrotron light sources worldwide.

While reports of transuranium experiments are generally scarce, we want to mention several beamlines that have started to develop their capabilities towards *L*_3_-edge X-ray emission spectroscopy experiments of various uranium or thorium samples. Pioneering work was carried out for intermetallic U compounds by Rueff *et al.* (2007 ▸) at ID16, and for U oxide compounds by us and collaborators at ID26 of the European Synchrotron Radiation Facility (ESRF) (Vitova *et al.*, 2010 ▸) and at the INE-Beamline, KIT Light Source [Karlsruhe Research Accelerator (KARA) storage ring] (Walshe *et al.*, 2014 ▸). Different types of three-analyzer-crystal X-ray emission spectrometers were used for U *L*_3_-edge studies at BL39XU at SPring-8 (Kawamura *et al.*, 2020 ▸; Honda *et al.*, 2020 ▸) and at beamline ID20 at Diamond Light Source (Pan *et al.*, 2020 ▸), and for Th *L*_3_-edge experiments at BL14W1 at the Shanghai Synchrotron Radiation Facility (SSRF) (Bao *et al.*, 2018 ▸; Duan, Bao *et al.*, 2017 ▸; Duan, Gu *et al.*, 2017 ▸). The microXAS beamline at the Swiss Light Source (SLS) started experiments with high spatial resolution (1 µm) using an emission spectrometer with cylindrical von Hamos geometry (Szlachetko *et al.*, 2012 ▸) at the U *L*_3_-edge (Grolimund, 2021 ▸). Moreover, a portable single-analyzer-crystal X-ray emission spectrometer was used at BL11-2 at the Stanford Synchrotron Radiation Laboratory (SSRL) to obtain U *L*_3_-edge X-ray emission spectroscopy data (Ditter *et al.*, 2020 ▸).

Concerning transuranium elements, early work has been performed at the high-resolution X-ray emission spectrometer (HR-XES) at beamline ID16 of the ESRF, where already in 2010 Am *L*_3_-edge resonant inelastic X-ray scattering (RIXS) data were recorded (Heathman *et al.*, 2010 ▸). Another beamline facility equipped to temporarily perform radionuclide work is beamline 6-2 at the SSRL. Here, a seven-analyzer-crystal Johann-type X-ray emission spectrometer with 1 m bending radius was used, *e.g.* to study the Pu *L*_3_-edge of intermetallic plutonium phases (Booth *et al.*, 2012 ▸, 2014 ▸), PuO_2_ (Tobin & Shuh, 2015 ▸) and the Np *L*_3_-edge for NpSe_2_ solid phases (Jin *et al.*, 2019 ▸). Popa *et al.* (2015 ▸) analyzed the structure of Pu(III) phosphate, applying the Johann-type X-ray emission spectrometer at the MARS beamline at SOLEIL for Pu and Am *L*_3_ HR-XANES measurements, and herewith confirming the +3 valence state of Pu as well as that of its Am daughter resulting from β^−^ decay. At the INE-Beamline in 2014, in a collaborative project with the Joint Research Center Karlsruhe, we investigated the oxidation states of Pu at the Pu *L*_3_-edge in a study exploring the phase diagram of UO_2_-PuO_2_ at high temperatures (Böhler *et al.*, 2014 ▸) and for Pu oxide nanocrystals (Hudry *et al.*, 2014 ▸).

A large increase in sensitivity to differences in the electronic structure unfolds for HR-XANES spectroscopy at the An *M*_4,5_-edges (Butorin *et al.*, 1996 ▸; Rothe *et al.*, 2012 ▸; Kvashnina *et al.*, 2013 ▸; Vitova *et al.*, 2013 ▸). Difficulties arise because the corresponding absorption and emission energies between ∼3 and 4.5 keV belong to the tender X-ray region. At these energies, air molecules as well as containment materials strongly scatter and/or absorb the X-rays [incident beam impinging onto the sample, X-ray fluorescence/inelastically scattered radiation emitted by the sample, and monochromated radiation diffracted and focused by the analyzer crystal(s) onto the detector]. For this reason, a specific adaption of the experimental stages, such as He-filled bags bridging the air gap between sample, crystal(s) and detector, an He encasement enclosing the whole spectrometer or even vacuum conditions, is required to enable efficient X-ray emission spectroscopy. Up to now, this has limited the reported *M*-edge studies of transuranium elements to only four beamlines worldwide. At the KIT Light Source a five-analyzer-crystal Johann-type X-ray emission spectrometer based on an original ID26 design was initially commissioned and successfully tested at the INE-Beamline for radionuclide research (Prüßmann, 2016 ▸; Vitova *et al.*, 2017 ▸; Bahl *et al.*, 2017 ▸; Popa *et al.*, 2016 ▸; Rothe *et al.*, 2012 ▸), and was later on transferred to the ACT station of the new CAT-ACT wiggler beamline after completion of the endstations in 2016 (Zimina *et al.*, 2017 ▸; Rothe *et al.*, 2019 ▸). With this spectrometer a large contribution to the development of the *M*-edge spectroscopy for the trans­uranium elements was achieved, *e.g.* the first-ever measured Pu *M*_5_-edge XANES/HR-XANES (Rothe *et al.*, 2012 ▸) was already published in 2012. Later on, the first Pu and Np *M*_5_-edge HR-XANES and 3*d*4*f* RIXS (Vitova *et al.*, 2017 ▸) and various Np, Pu, Am and U *M*-edge HR-XANES spectra were recorded (Vitova *et al.*, 2017 ▸, 2020 ▸; Bahl *et al.*, 2017 ▸; Epifano *et al.*, 2019 ▸). At the MARS beamline of the SOLEIL synchrotron – belonging to the few dedicated synchrotron radiation stations for the investigation of nuclear materials including waste forms – an He-filled bag between the sample position, a single analyzer crystal with 1 m bending radius in Johann geometry and the detector can be used to reduce absorption in air. With this setup, Pu *M*_4_-edge HR-XANES/RIXS experiments on plutonium carbonate samples (Pidchenko *et al.*, 2020 ▸), PuO_2_ phases (Gerber *et al.*, 2020 ▸) and uranium compounds were performed (Hunault *et al.*, 2019 ▸). At beamline ID26 of the ESRF a five-analyzer-crystal Johann-type X-ray emission spectrometer with 1 m bending radius was employed to record, for example, the Pu *M*_4_-edge of plutonium nanoparticles (Gerber *et al.*, 2020 ▸; Kvashnina *et al.*, 2019 ▸). A similar spectrometer is meanwhile situated at the ESRF Rossendorf beamline (BM20 ROBL), another dedicated beamline for radionuclide research. Although only *L*_3_-edge data of Pu, U and Th have been published so far (Amidani *et al.*, 2021 ▸; Gerber *et al.*, 2020 ▸), the whole spectrometer can be placed inside a bag filled with He to record edges at tender energies.

Besides applying crystal spectrometers in the tender to hard X-ray spectral range, a few beamlines worldwide took provisions for high-resolution An spectroscopy research in the soft X-ray to vacuum ultraviolet range, giving access to shallower absorption edges – with naturally narrow line widths – at the An *N* or *O* levels (or the core levels of low-*Z* ligand atoms) or to hard X-ray inelastic scattering with meV resolution range, enabling the detection of specific X-ray photon–phonon interactions, *i.e.* accessing basic solid-state properties such as superconductivity in solid-state An materials. In the former research area, pioneering An spectromicroscopy work employing the soft X-ray scanning transmission X-ray microscopy endstation at Advanced Light Source (ALS) beamline 11.0.2 (Lawrence Berkeley National Laboratory) has been reported (Dalodière *et al.*, 2017 ▸). Scientists employing ALS beamline 7.0.1’s soft X-ray absorption and resonant scattering capabilities have provided, for example, Pu *N*_6,7_-edge XANES data as well as NpO_2_ RIXS at the Np *O*_5_-edge (∼100 eV) (*cf*. Modin *et al.*, 2011 ▸; Tobin *et al.*, 2002 ▸; Butorin *et al.*, 2013 ▸, 2016 ▸). Feasibility of experiments in the latter field has been demonstrated, for example, in Pu metal/PuO_2_ scattering experiments at the Advanced Photon Source (APS) beamline 30-ID-B,C (HERIX endstation, Argonne National Laboratory), offering momentum-resolved inelastic X-ray scattering with high resolution (∼1.5 meV) (Manley *et al.*, 2009 ▸, 2012 ▸).

Almost all of the reported HR-XES studies work with relatively high An concentrations. This, however, often prevents studies of environmental samples from contaminated land sites or sorption and diffusion samples from experiments in the context of safety case studies in nuclear waste disposal research, where loadings of actinides below the p.p.m. range should prevail. In this article, we present the technical developments at the ACT station towards enabeling those low An loading HR-XANES experiments while still having the flexibility for other experimental techniques such as conventional high-energy X-ray absorption fine structure (XAFS) in transmission or total fluorescence-yield detection mode and Laue-type high-energy or wide-angle X-ray scattering (HEXS/WAXS) up to ∼55 keV photon energy.

As aforementioned, another important point is sample integrity upon irradiation conditions at highly brilliant synchrotron radiation sources. Different types of beam-induced alterations are observed at ambient conditions, especially for often redox-labile An specimens (Wilk *et al.*, 2005 ▸; Denecke *et al.*, 2005 ▸). These photo-oxidations or photo-reductions mostly occur for aqueous systems or hydrated pastes and, thus, are especially relevant for environmental samples. Beam-induced changes need to be thoroughly monitored and excluded at the utmost degree to obtain unbiased speciation information. One possibility to exclude those changes is cooling the samples below 180 K (Göttlicher *et al.*, 2018 ▸). From screening the relevant literature, to the best of our knowledge, all ‘photon hungry’ HR-XANES/XES experiments on transuranium elements up to now have been performed at room temperature. In Section 3 we will present the development of a cryogenic sample holder for radioactive samples adapted to a commercial liquid N_2_/He cryostat. Here the challenge was to design and approve a system for double encapsulation featuring sufficiently X-ray transparent windows for spectroscopy in the tender X-ray region while – at the same time – withstanding thermal-isolation vacuum conditions.

## 2. INE beamline facilities at the KIT Light Source

INE at KIT (KIT-INE) operates two experimental stations dedicated to the investigation of radionuclide materials by X-ray based methods at KARA (former ANKA synchrotron light source) – the INE-Beamline at a bending magnet port (fully operational since 2005) and the ACT laboratory at the CAT-ACT wiggler beamline (commissioned in 2016) (Rothe *et al.*, 2012 ▸, 2019 ▸; Zimina *et al.*, 2017 ▸). Both beamline hutches are equipped and licensed to investigate radioisotopes up to activities equal to 10^6^ times the (European) exemption limits and 200 mg for the fissile isotopes ^235^U or ^239^Pu (the exemption limits are generally 10^3^ or 10^4^ Bq for most relevant radionuclides under investigation at these beamlines). They are permanently designated as monitored areas for handling radioactive materials. Their status can be upgraded to temporary controlled areas whenever radionuclide inventories exceed the exemption limits (applying a sum rule in the presence of multiple isotopes). The license at both stations enables the investigation of ‘hot’ materials including genuine nuclear-waste forms as well as *in situ* investigations at non-ambient conditions (*e.g.* high *p* and/or high *T*) of radionuclides within a double containment. The beamline concept benefits from the flexibility of evaluating and approving new experimental setups by INE’s own technical commission, ensuring adherence to safety regulations while at the same time avoiding the limitation of experiments by standardized sample containments or an α-box environment. The focus at both beamlines has been originally placed on XAFS-based speciation investigations in the context of the nuclear-waste-disposal safety case (encompassing processes during interim storage of spent nuclear fuel or nuclear-waste glass and final deep geological disposal of these materials). More recently, another emphasis has been placed on fundamental An studies exploiting the capabilities of HR-XES techniques within the basic KIT/Helmholtz research (NUSAFE program topic) or third-party-funded projects such as the European Research Council (ERC) Consolidator grant ‘THE ACTINIDE BOND properties in solid, liquid and gas state’.

## 3. Recent upgrades at the ACT station

**Figure 1:**
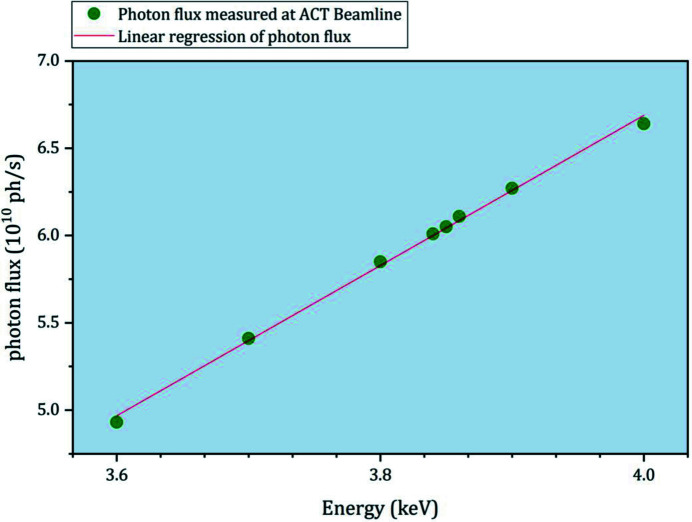
Current photon-flux conditions in the tender X-ray energy range at the ACT experiment. Synchrotron radiation beam path conditions from source to *I*_0_ monitor (ionization chamber): 2.5 GeV storage-ring electron energy, average electron-beam current ≃ 120 mA, 2 mm (h) × 1 mm (v) front-end (white beam) slit aperture, cylindrical collimating first Si mirror, 250 µm Be vacuum protection plus 100 µm graphite thermal-protection window, Si〈111〉 DCM crystal pair, toroidal focusing second Si mirror, 25 µm KAPTON window and synchrotron radiation beam spot size ≃ 1 mm × 1 mm.

**Figure 2:**
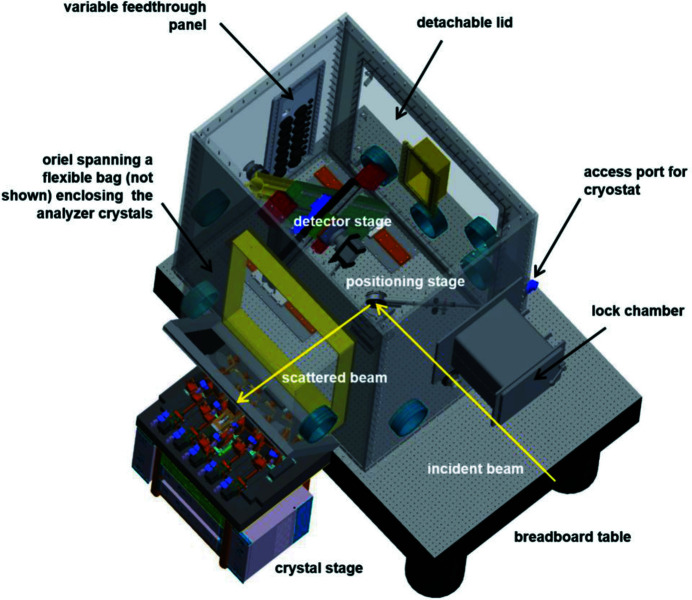
A 3D CAD model of the He box, providing an improved concept with possibilities for several experiments, *e.g.* enabling *in situ* measurements and cryostat experiments at the *M*-edges of actinides at the ACT station.

The Johann-type X-ray emission spectrometer at the ACT laboratory is routinely applied for HR-XES/HR-XANES and RIXS experiments in a broad energy range encompassing the An *M*- and *L*-edges (Th–Es feasible). Several analyzer-crystal sets [five each of Si(111), Si(110) and Ge(111), and four each of Ge(220) and Ge(311), Saint-Gobain, France] with a bending radius of 1 m are available to cover the relevant absorption levels. The tender X-ray range at ACT is accessible with an Si〈111〉 crystal pair in the cryo-cooled double-crystal monochromator (DCM) down to ∼3.4 keV [*cf*. Zimina *et al.* (2017 ▸) for details]. The beam is focused by a toroidal Si mirror, resulting in a spot size of ∼1 mm × 1 mm. The photon flux between 3.6 and 4 keV shown in Fig. 1 ▸ has been precisely determined using a short ionization chamber (Oken, Japan, model S-1329A1 with 33 mm electrode length) filled with N_2_ at ambient pressure. In the tender X-ray region, scattering and absorption of X-rays is efficiently minimized by enclosing all beam paths – *i.e.* that of the impinging beam and those between the sample, the analyzer crystals and the single-diode silicon-drift-detector entrance window (KETEK VITUS SDD, Germany) arranged in a vertical Rowland circle geometry – in He atmosphere. A rigid plexiglass box housing the spectrometer components has been designed and recently installed on the ACT breadboard table (Fig. 2 ▸). The improved design allows one to keep stable conditions with less than 150 p.p.m. oxygen inside the box through a controlled He flow of ∼5 l min^−1^. The five crystal holders are placed inside a flexible polyvinyl chloride (PVC) bag clamped by the five crystal mounts and spanned by an oriel protruding from the left-hand side wall (in beam direction) of the He box. This setup allows for sufficient freedom of motion of the crystals along their individual Rowland circles. The He box is equipped with a spacious rectangular lock chamber for exchanging sample cells (*e.g.* *in situ* cells or combined UV–Vis/XES setups) and a panel at the back side (in beam direction) providing various media and power feedthroughs (*e.g.* He/N_2_/Ar inert gas supply, cooling water, motor power/encoder/limit-switch lines, detector high-voltage/power supply and signal lines, vacuum pump hose, *etc*.). A special access port based on a gear-stick sleeve at the front-side right-hand corner (in beam direction) of the box allows one to insert the supply lines for the modified LN_2_ cryostat, which are bundled in a flexible stainless-steel tube (*cf*. Fig. 5, left image, and the detailed description below). The plexiglass box is further equipped with a large detachable lid sealed by a PVC gasket at the wall opposite from the crystal stage (right-hand side in beam direction). The large opening provides inside access for installation of the standard transmission/fluorescence XAFS detection equipment with ionization chambers (Poikat, Germany, positioned on X-95 rails) and an electrically cooled eight-element LEGe detector (Mirion, France, *cf*. Fig. 5, right image) or a Laue diffraction setup using X-ray sensitive storage screens (PerkinElmer, USA). In closed configuration at inert gas conditions, long-sleeved gloves at various positions at all four side walls and the oriel allow one to handle sample cells and manipulate samples and equipment inside the box, including the exchange of analyzer-crystal sets.

Compared with the original setup described by Zimina *et al.* (2017 ▸), the improved design of the box enclosing the HR-XES setup offers the following advantages:

(i) The box remains permanently installed on top of the breadboard table, minimizing the time to switch between HR-XES and standard X-ray absorption spectroscopy (XAS) experiments at ACT.

(ii) The front and back side walls of the box are fitted with ISO-KF 50 flange feedthroughs, simplifying switching between ACT and CAT stations by bridging the box with a vacuum pipe (possible without opening the box).

(iii) There is significantly improved He gas purity and correspondingly higher photon flux.

(iv) There is significantly reduced He consumption during tender X-ray measurements.

(v) It has a large lock chamber, *e.g.* for transfer of *in situ* sample cells.

## 4. Implementation of a liquid nitro­gen flow-through cryostat for HR-XES measurements

**Figure 3:**
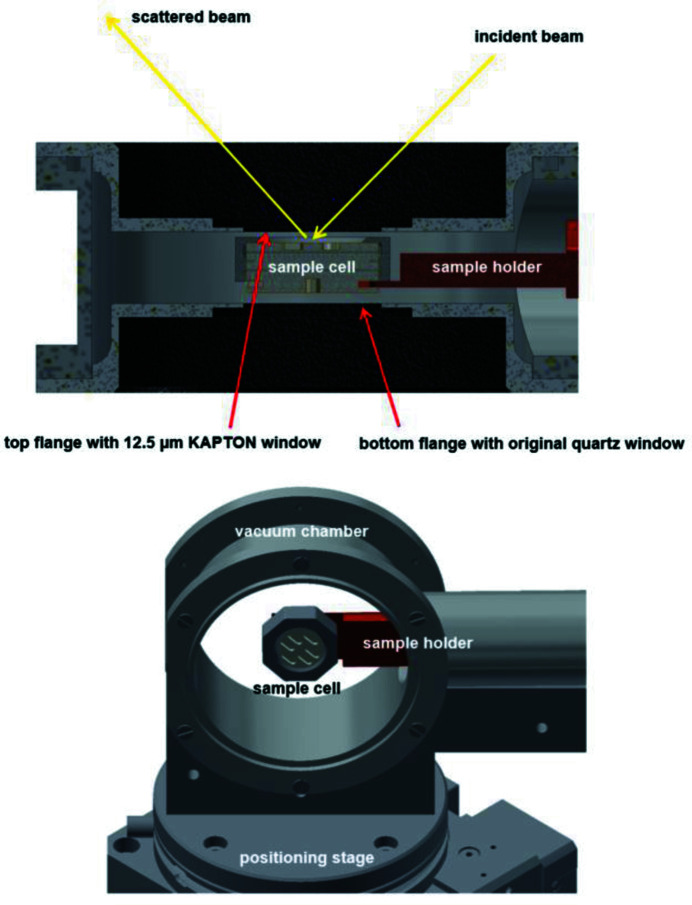
Top – a cross-section CAD drawing of the modified MicrostatHe vacuum chamber (side view). The original sample holder has been replaced by a copper fork clamping the sample cell, as described in Section 4. Bottom – a 3D CAD drawing of the vacuum chamber with removed flanges mounted on top of the positioning stage, exhibiting the sample holder (copper fork) and sample cell assembly.

**Figure 4:**
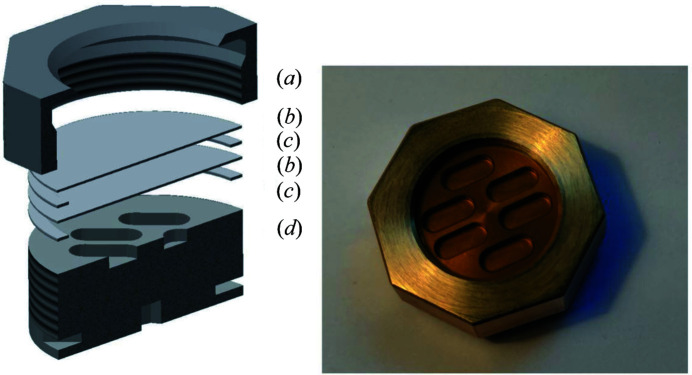
Left image – an exploded-view 3D CAD drawing of the sample cell assembly. From top to bottom: (*a*) cap nut, (*b*) KAPTON disk, (*c*) TEFLON gasket, (*b*) KAPTON disk, (*c*) TEFLON gasket, (*d*) cell body. Right image – a top view of a sealed sample cell with six sample cavities (outer diameter ≃ 30 mm).

**Figure 5:**
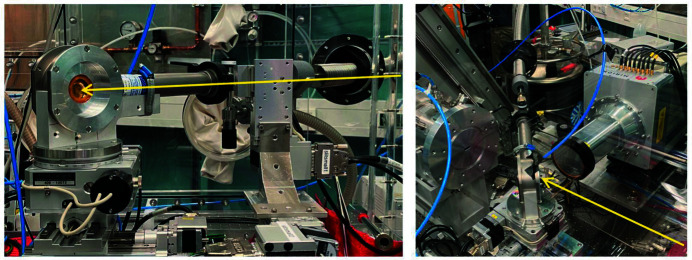
Left image – the cryostat vacuum chamber rigidly mounted on top of the sample positioning stage (analyzer-crystal point of view). The sample cell is visible through the outer KAPTON vacuum window. The arrow marks the incoming beam passing the auxiliary four-blade slit in front of the sample. Right image – the cryostat used inside the He box with opened lid for standard fluorescence XAFS detection mode. The arrow marks the incoming beam.

A commercial flow-through cryostat primarily developed for microscopy applications (Oxford Instruments MicrostatHe, UK) – optionally operational with LHe or LN_2_ as cryogenic coolant – was selected to be adapted to the HR-XES setup at ACT. It has been modified for tender X-ray (An *M*-edge) requirements while providing a special clamp mechanism enabling fast sample changes with the new cryo-sample cells. The instrument was chosen based on the special vacuum-chamber dimensions offering a large solid-angle field of view (∼140° opening angle) onto the sample(s) and a narrow gap of ∼2 mm between the sample surface and the outer vacuum window (as required, *e.g.* for cryo-microscopy investigations). This in turn allows the X-rays isotropically emitted from the sample to be captured and diffracted by all five analyzer crystals in the 1 m Rowland circle arrangement. The original cryostat sample holder – bolted to the heat-exchanger block with the liquid coolant circulating inside absorbing the thermal energy – was replaced by a copper fork with a slot clamping the actual sample cells (Fig. 3 ▸). The sample cell (Fig. 4 ▸) – adhering to the double containment rule for radioactive samples – consists of six stacked components (from bottom to top): the threaded anodized aluminium body with a groove for the copper fork and up to six elongated cavities milled into one side receiving different sample materials (solids/powders, wet pastes or liquids), a flat ring-shaped TEFLON gasket, an 8 or 12.5 µm KAPTON (polyimide) disk, a second TEFLON gasket, a second KAPTON disk, and a brass cap nut with a large opening giving access to the sample cavities below the transparent KAPTON membranes. The nut is tightly screwed onto the cell body, pressing the stacked windows and gaskets against each other, the disk and the cap on top and thereby tightly encapsulating the radioactive materials. Extensive pumping tests exposing the sealed sample cell to the thermal-insulation vacuum (in the 10^−5^ mbar range) have been carried out with inactive dummy samples prior to initial experiments with radioactive materials. The loaded sample cells are pre-frozen in an LN_2_ bath and introduced via the lock chamber into the dry He atmosphere inside the box. The cylindrical cryostat chamber is opened at cryogenic temperatures and the sample cell is attached to the copper fork. The original quartz window of the sample chamber flange facing the top side of the sample cell has been replaced by an ep­oxy-glued 12.5 µm KAPTON disk. Although already absorbing ∼35% of the photon intensity at the Np *M*_5_-edge energy [*E* Np(3*d*_5/2_) ≃ 3664 eV, *E* Np(*M*_α1_) ≃ 3261 eV], the cryostat window thickness has been a necessary compromise between vacuum stability and X-ray transparency. The vacuum chamber attached to the flexible tube containing the LN_2_ supply and exhaust lines, as well as thermal sensor (PT-100 type) and heater connections, is mounted by a half-shell adapter on top of the sample positioning stage at 45° relative to the impinging beam (Fig. 5 ▸, left image). The sample *x*-/*y*-/*z*-position is precisely adjusted by a set of crossed alignment lasers. A modified sample cell was fitted with a second PT-100 sensor to measure the temperature directly at one of the sample cavities. This setup enabled a sample temperature of 141.2 ± 1.5 K at irradiation conditions with the heat-exchange block cooled down and stabilized at LN_2_ temperature (∼77 K). So far no attempts to operate the cryostat equipment with liquid He as coolant have been made. Future design modifications aim at improving the thermal contact between sample cell and clamping mechanism.

## 5. Np *M*_5_-edge HR-XANES measurements at low concentrations and cryogenic conditions

As already mentioned above, oxidation-state changes of redox-sensitive An elements [primarily U, Np and Pu, which may exist (or even co-exist) at different oxidation states (IV–VI) at the relevant geochemical conditions] are not exclusively induced by redox partners such as Fe(II) in mineral surface reactions. These changes have been observed to occur for mostly wet samples in XAS-based speciation experiments under inert gas conditions. It is suspected that water radicals formed in bright X-ray beams might interact with the An cations and change their oxidation state. Another conjecture is effects due to increased sample temperatures at X-ray irradiation conditions. As an example to illustrate the performance of our improved tender X-ray emission spectroscopy setup, we present Np *M*_5_-edge HR-XANES results in relation to our recent study on the interaction mechanisms of ^237^Np – a long-lived α-emitting isotope generated during operation of nuclear fission reactors – with clay minerals, which are highly relevant sorbents in the multi-barrier concept for nuclear-waste disposal in deep-geological formations. In this context, illite is considered as an important mineral fraction of several clay formations discussed as potential host rocks. Details on the successful measurement of low Np concentrations on illite are currently under revision in a previously submitted article (Schacherl *et al.*, 2021 ▸). Therein, experiments at conditions expected to prevail in the far field of a breached disposal site, *i.e.* low Np(IV/V) concentrations down to 1 p.p.m., are described in order to verify the lowest possible Np loadings on clay surfaces for which Np speciation using the HR-XANES technique is still possible. Moreover, in order to suppress radiation-induced changes, the samples in our Np *M*_5_-edge HR-XANES experiments were cooled down to 141.2 ± 1.5 K using the setup described above. These results were subsequently compared with room-temperature measurements.

An Illite du Puy (6.94% Fe_2_O_3_) (Montoya *et al.*, 2018 ▸) sample was contacted with Np at an initial concentration of *c*_0_[Np(V)] = 1 × 10^−6^ mol l^−1^ for 11 days at pH 9.2 with a solid-to-liquid ratio of 2 g l^−1^ and *I* = 0.1 mol l^−1^ NaCl, resulting in a sorbed Np loading on illite of 83 ± 2 p.p.m. The sample preparation procedure is as well described in detail by Schacherl *et al.* (2021 ▸). After centrifugation at 15000 r min^−1^ for 80 min (LLG-uniCFUGE 5, Lab Logistics Group GmbH, Germany), the wet illite paste was transferred to the cryostat sample cell in an intert gas (Ar) glove box and encapsulated by two 8 µm KAPTON layers (polyimide film, Advent Research Materials, United Kingdom). The sealed sample cell was checked for the absence of surface contamination and transferred to the beamline inside a gastight transport container. At the ACT station the sample cell was pre-frozen in a liquid nitro­gen bath and subsequently mounted at the sample holder inside the He box as described in Section 4.

A solid Np reference sample {K_3_Na[U,NpO_2_(CO_3_)_3_] × H_2_O (Vitova *et al.*, 2020 ▸)} was used to calibrate the DCM for Np *M*_5_-edge HR-XANES spectroscopy. The maximum of the most intense absorption resonance (‘white line’) of this sample was assigned to 3664 eV. The Np *M*α_1_ fluorescence line was recorded with four Si〈220〉 analyser crystals, aligned at a Bragg angle of 81.92°. An energy range from 3658 to 3674 eV with a step size of 0.2 eV across the edge (3660–3666 eV) and 0.4 eV in the pre- and post-edge region was scanned for every spectrum. For the sample at room temperature, 9 × 10 min scans were recorded. For the sample mounted inside the cryostat at 141.2 ± 1.5 K, 9 × 15 min scans were recorded. The program *OriginPro* (OriginLab, 2018 ▸) was used to calibrate the spectra using the reference scans recorded before and after each sample scan.

**Figure 6:**
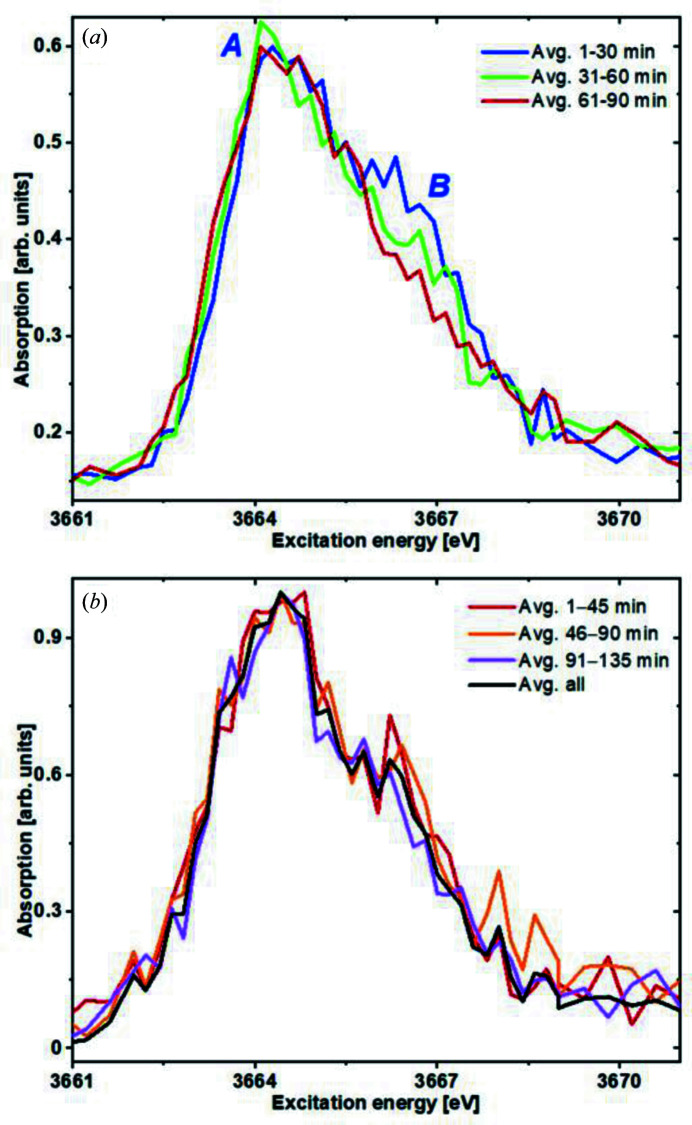
(*a*) Gradual change of Np *M*_5_-edge HR-XANES spectra of a sample with 83 ± 2 p.p.m. Np sorbed on illite (*cf*. Section 6 for sample details) at 300.0 ± 1.5 K. Changes in the shoulder denoted as feature B are visible with irradiation time. (*b*) At cryogenic conditions (141.2 ± 1.5 K), no significant changes of the spectra are discernible within the noise level.

The higher photon flux achieved with the advanced He box setup, further optimization of the beamline optics alignment and recent improvements of the KARA storage ring operation have led to the observation of beam-induced alterations in hydrated samples such as wet Np-sorbed illite pastes. This is clearly shown in Fig. 6 ▸(*a*), where the average of several Np *M*_5_-edge HR-XANES scans of the Np/illite sample with the progression of irradiation time of a series of measurements performed at room temperature on the same sample spot are depicted. Peak *B* – significant for the presence of Np(V) ‘neptunyl’ species (Vitova *et al.*, 2020 ▸) – disappears after prolonged irradiation time, strongly suggesting reduction of Np(V) to Np(IV). The ‘white line’ maximum (peak *A*) position does not significantly change upon reduction of Np(V) to Np(IV) – a well known anomaly for pentavalent ‘actinyl’ species upon the loss of the *trans*-dioxo conformation upon transition to the tetravalent state (Vitova *et al.*, 2015 ▸, 2017 ▸, 2018 ▸, 2020 ▸; Podkovyrina *et al.*, 2016 ▸).

In contrast to that, it becomes clearly evident from the comparison of scans averaged for different time intervals, as depicted in Fig. 6 ▸(*b*), that beam-induced reduction is successfully suppressed when applying the cryostat setup stabilizing a sample temperature of 141.2 ± 1.5 K. Within the noise level, no significant changes in the spectra were detected when irradiating the same spot for up to more than 130 min.

## 6. Outlook

Several technical upgrades and extensions at the ACT station are foreseen to be realized in the near future. Some of them have been already specified and are in the state of procurement:

(1) A single-element SDD (Hitachi Vortex-EX60, USA) will be added to the detector pool to allow for standard (total) fluorescence-yield XAFS spectra recorded parallel to the HR-XANES measurements. This device will facilitate the precise localization of sample coordinates in the case of multiple-position sample holders, *e.g.* the cryo-cells described in Section 4.

(2) The flexible reusable storage phosphor screens used so far for Laue-type diffraction (WAXS/HEXS) experiments (*cf*. *e.g.* Bouty *et al.*, 2021 ▸) read out by a laser scanner (Perkin­Elmer Cyclone Plus, USA) following each exposure – a time-consuming procedure preventing the investigation of dynamic processes – will be replaced by an X-ray camera with a large active area (164 mm-diameter fluorescence screen) with a tapered waveguide (Photonic Science sCMOS_37MP, United Kingdom).

(3) Based on the excellent performance at INE-Beamline, a new set of mono- and poly-capillary X-ray lenses (Helmut Fischer GmbH, Germany), covering the tender to hard X-ray range, has been ordered to collimate or focus the impinging X-rays, increasing angular resolution and flux density for Laue-type diffraction measurements as well as enabling spatially resolved µ-XAFS/XRF (optionally in confocal detection mode) and µ-HR-XANES measurements with spot sizes down to 10 µm (FWHM). The capillary optics will be positioned and precisely aligned in the beam using a hexapod microrobot (Physik Instrumente H-811, Germany).

Five years after its initial commissioning, the ACT station at the CAT-ACT wiggler beamline has been developed into a unique X-ray spectroscopy station for the investigation of various radionuclide materials with state-of-the-art speciation techniques – strongly focusing on ‘flux hungry’ photon-in/photon-out techniques. The outlined modifications and improvements will even increase the high flexibility and sensitivity in the near future. The ACT station – although officially situated at a KIT in-house large-scale research facility – may be accessed by external users through direct cooperation with KIT-INE (https://www.ine.kit.edu).
